# Biogeography of the Shimba Hills ecosystem herpetofauna in Kenya

**DOI:** 10.24272/j.issn.2095-8137.2017.048

**Published:** 2018-03-07

**Authors:** Patrick K. Malonza, David M. Mulwa, Joash O. Nyamache, Georgina Jones

**Affiliations:** 1 Section of Herpetology, National Museums of Kenya, Nairobi 40658-00100, Kenya; 2 Base Titanium Ltd, Ukunda 1214-80400, Kenya; 3 Sino-Africa Joint Research Center, Chinese Academy of Sciences, Nairobi 40658-00100, Kenya

**Keywords:** Amphibians, Reptiles, Eastern Africa coastal forests, Eastern Arc Mountains, Indicator species, Zoogeography

## Abstract

The Shimba Hills ecosystem along the south coast of Kenya is a key East African biodiversity hotspot. Historically, it is biogeographically assignable to the East African coastal biome. We examined the current Shimba Hills herpetofauna and their zoogeographical affinities to the coastal forests and nearby Eastern Arc Mountains biodiversity hotspots. The key studied sites included the Shimba Hills National Reserve, forest reserves, Kaya forests, and adjacent private land. Data on herpetofaunal richness were obtained from recent field surveys, literature, and specimens held at the National Museums of Kenya, Herpetology Section Collection, Nairobi. The Makadara, Mwele, and Longo-Mwagandi forests within the Shimba Hills National Reserve hosted the highest number of unique and rare species. Generally, the forest reserves and Kaya forests were important refuges for forest-associated species. On private land, Mukurumudzi Dam riparian areas were the best amphibian habitat and were host to three IUCN (Red List) Endangered-EN amphibian species, namely, *Boulengerula changamwensis, Hyperolius rubrovermiculatus*, and *Afrixalus sylvaticus*, as well as one snake species *Elapsoidea nigra*. Using herpetofauna as zoogeographic indicators, the Shimba Hills were determined to be at a crossroads between the coastal forests (13 endemic species) and the Eastern Arc Mountains (seven endemic species). Most of the Eastern Arc Mountains endemic species were from recent records, and thus more are likely to be found in the future. This ‘hybrid’ species richness pattern is attributable to the hilly topography of the Shimba Hills and their proximity to the Indian Ocean. This has contributed to the Shimba Hills being the richest herpetofauna area in Kenya, with a total of 89 and 38 reptile and amphibian species, respectively. Because of its unique zoogeography, the Shimba Hills ecosystem is undoubtedly a key biodiversity area for conservation investment.

## INTRODUCTION

Biogeography is the study of the geographic distribution of organisms, and includes historical, ecological, and conservation biogeography. Historical biogeography addresses the causes of the past distribution of species, whereas ecological biogeography studies the factors (mainly biotic and abiotic) that define the current spatial distribution of species (Monge-Nájera, 2008). Conservation biogeography is the application of biogeographical principles, theories, and analyses, especially on species distribution to problems concerning biodiversity conservation (Whittaker et al., 2005).

Global biodiversity hotspots are areas that support high species richness and endemism, and face considerable threats relative to the remaining area (Mittermeier et al., 2005; Myers et al., 2000, 2003). Currently, there are 35 global biodiversity hotspots, with two represented in Kenya (Sloan et al., 2014). One is the Eastern Afromontane hotspot represented by the Taita Hills plus mountain and highland areas in central (Mt. Kenya, Aberdare Range, and Nyambene Hills) and western (Kakamega Forest, Nandi Forests, Cherangani Hills, and Mau Hills) Kenya. The other is the coastal forests of eastern Africa represented by the coastal forests of Kenya, with the two most well-known being the Arabuko-Sokoke Forest and Shimba Hills. 

Historically, the Shimba Hills have been categorized as a coastal forest (Burgess & Muir, 1994; Howell, 1993) and are one of the richest herpetological areas in Kenya. Other rich herpetofaunal areas include the Arabuko-Sokoke Forest (Chira, 1993; Drewes, 1992); Kakamega Forest (Wagner et al., 2008; Lötters et al., 2007;; Wagner & Böhme, 2007; Schick et al., 2005), and Taita Hills (Malonza & Veith, 2012; Malonza et al., 2010; Burgess et al., 2007; Malonza, 2008; Newmark, 2002; Lovett, 1990). 

The Shimba Hills ecosystem has diverse habitat types that include forests, woodlands, grasslands, bushlands, and wetlands (rivers, swamps, and dams). This habitat diversity relates to the habitat heterogeneity hypothesis, which suggests that structurally complex habitats provide more niches and diverse ways of exploiting environmental resources and hence increase species diversity (Tews et al., 2004). In the Shimba Hills ecosystem, some of the key amphibian and reptile habitat areas are manmade dams such as Mukurumudzi. In addition to the gazetted forest reserves are the sacred Mijikenda community Kaya forests, which vary from small to large and are key sites that act as refuges for unique and rare biodiversity within community land (Githitho, 2003). The flagship herpetofaunal species for the Shimba Hills ecosystem are the endemic Shimba Hills reed frog *Hyperolius rubrovermiculatus* Schiøtz, 1975, and *Afrixalus sylvaticus* (Schiøtz, 1974), which are listed as Endangered (EN) in the IUCN Red List. 

Past herpetological museum collections in the Shimba Hills have given an indication that this area might be a unique site, recording species both endemic to lowland coastal forests as well as the Eastern Arc Mountain forests. No past biodiversity work has assessed the exact biogeographical assignment of the Shimba Hills because it has been assumed to be a typical coastal forest. Recently, however, Bwong et al. (2014) examined some of the area’s amphibians and hinted that the Shimba Hills National Reserve might be at a biogeographical crossroads. 

Therefore, the aim of this paper was to assess the amphibian and reptile fauna of the Shimba Hills ecosystem, with a focus on their zoogeographical affinities. Herpetofauna are good zoogeographical indicators due to their low dispersal abilities compared with higher vertebrates like birds and mammals (Bell & Donnelly, 2006; Pineda & Halffter, 2004). We used herpetofauna to test the hypothesis that the Shimba Hills ecosystem is a coastal forest.

## MATERIALS AND METHODS

### Study area

**Figure 1:**
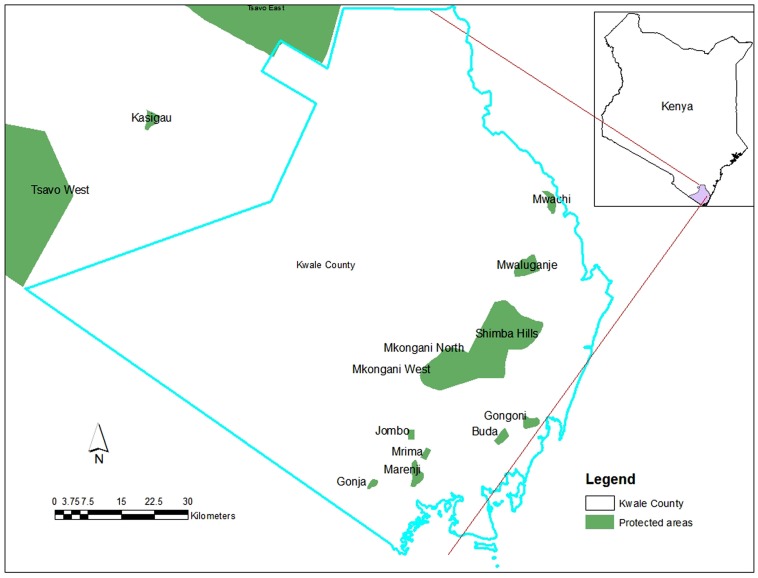
Map of Kwale showing the Shimba Hills National Reserve, Buda, Gongoni and other protected forest reserves (Drawn by Edwin N. Gichohi)

The Shimba Hills ecosystem occurs along the south coast of Kenya in Kwale County (Figure 1). The ecosystem is diverse and dotted with important biodiversity sites ranging from forests, bushlands, and grasslands to wetlands, and all at varying levels of habitat disturbance. These sites occur from sea level up to about 430 m in hills within the Shimba Hills National Reserve. The Shimba Hills ecosystem includes protected areas such as the Shimba Hills National Reserve, Buda Forest, Gongoni Forest, Mwaluganje Community Wildlife Conservancy, Kaya forests, community lands, and private dams such as Mukurumudzi. 

### Herpetofaunal sampling

Field sampling was conducted in different sites covering varying seasons from July 2012 to June 2017. Each sampling visit was done for two weeks covering different sites. The periods and frequencies of sampling visits are provided below:

1. July/August 2012 (dry season) Mukurumudzi River Dam site (Miembeni, Nguluku, and Maumba);
2. November/December 2012 (wet season) Mukurumudzi River Dam site (Miembeni, Nguluku, and Maumba);
3. April/May 2013 (dry/wet season) Mukurumudzi River Dam site (Miembeni, Nguluku, Maumba, and dam-site);
4. June 2013 (wet season) Mukurumudzi Dam (Miembeni, Nguluku, Maumba, and dam-site), Mtawa stream, and Gongoni Forest pond;
5. September 2013 (dry season) Mukurumudzi Dam (Miembeni, Nguluku, Maumba, and dam-site);
6. November/December 2013 (wet season) Mukurumudzi Dam (Miembeni, Nguluku, Maumba, dam-site, and tailings storage facility-Vumbu), Gongoni Forest, and Gongoni Forest pond;
7. March/April 2014 (wet season) Mukurumudzi Dam (Miembeni, Nguluku, Maumba, dam-site, and tailings storage facility-Vumbu), Gongoni Forest, and Gongoni Forest pond;
8. June/July 2014 (wet season) Mukurumudzi Dam (Miembeni, Nguluku, Maumba, dam-site, and tailings storage facility-Vumbu);
9. September 2014 (dry season) Mukurumudzi Dam (Miembeni, Nguluku, Maumba, dam-site, and tailings storage facility-Vumbu);
10. November/December 2014 (wet season) Mukurumudzi Dam (Miembeni, Nguluku, Maumba, dam-site, and tailings storage facility-Vumbu);
11. March/April 2015 (dry season) Mukurumudzi Dam (Miembeni, Nguluku, Maumba, dam-site, and tailings storage facility-Vumbu);
12. May 2015 (wet season) Shimba Hills National Reserve (Longo Forest, Makadara Forest, Mwele Forest, Kaya Kwale Forest, Shimba Lodge wetland, Mwadabara bushland, Mwadabara wetland, Kivumoni Gate wetland, and Sheldrick Falls wetland), Gongoni Forest, Gongoni pond, Buda Forest, Mwabanda community land wetland, and Mukurumudzi Dam (Miembeni, Nguluku, Maumba, dam-site, and tailings storage facility-Vumbu);
13. August/September 2015 (wet season) Shimba Hills National Reserve (Longo Forest, Makadara Forest, Shimba Lodge wetland, and Mwadabara wetland), Gongoni Forest, Gongoni pond, Buda Forest, and Mukurumudzi Dam (Miembeni, Nguluku, Maumba, dam-site, and tailings storage facility-Vumbu);
14. November 2015 (wet season) Shimba Hills National Reserve (Longo Forest, Makadara Forest, Kaya Kwale Forest, and Shimba Lodge wetland), Gongoni Forest, Gongoni pond, Buda Forest, and Mukurumudzi Dam (Miembeni, Nguluku, Maumba, dam-site, and tailings storage facility-Vumbu);
15. March 2016 (dry season) Shimba Hills National Reserve (Longo Forest, Makadara Forest, and Shimba Lodge wetland), Gongoni Forest, Buda Forest, Mukurumudzi Dam (Miembeni, Maumba, Nguluku, dam-site, and tailings storage facility-Vumbu), Kaya forests (Kinondo, Ukunda, Tiwi, Muhaka, Waa, Sega, Lunguma, Chonyi, and Gandini);
16. May/June 2016 (wet season) Shimba Hills National Reserve (Longo Forest, Makadara Forest, Shimba Lodge wetland, and Mwadabara wetland), Gongoni Forest, Gongoni pond, Buda Forest, Mukurumudzi Dam (Miembeni, Maumba, dam-site, tailings storage facility-Vumbu), and Kaya forests (Kinondo, Muhaka, and Gandini);
17. December 2016 (dry season) Shimba Hills National Reserve (Longo Forest, Makadara Forest, and Shimba Lodge wetland), Gongoni Forest, Mukurumudzi Dam (Miembeni, Maumba, dam-site, and tailings storage facility-Vumbu), and Kaya forests (Kinondo and Muhaka);
18. June 2017 (wet season) Mukurumudzi Dam (Miembeni, Maumba, dam-site, and tailings storage facility-Vumbu), Shimba Hills National Reserve (Longo Forest, Makadara Forest, Mwele Forest, Shimba Lodge wetland, Mwadabara wetland, and Mwele Forest stream), Gongoni Forest, Gongoni pond, and Kaya forests (Kinondo and Muhaka).

Two standard methods (timed-species count and pitfall trapping) were used during field sampling.

(i) Time-limited searching (TLS): This is a form of timed-species counting. Here, sampling was conducted for a limited time period of two hours during the day and one hour at night within a given selected habitat without setting boundaries using three observers. The procedure is similar to the time constrained search-and-seize method described previously (Heyer et al., 1994; Karns, 1986). It involves quietly and slowly walking and recording all individual reptiles and amphibians found in different micro-habitats such as on the ground, in debris, under decomposing logs, on trees, shrubs, grass, reeds, and sedges, for one-person hour. It also involves digging in suitable micro-habitats in search of burrowing species such as skinks, caecilians, frogs, and snakes. For reptiles, this was done mostly in the morning session when they are most active, and can be observed basking, resting, or foraging. Night searches for amphibians involved visiting various selected wetlands (river beds/edges, ponds, pools, and marshlands) for one hour from 19:00hrs. In wetlands at night we used both visual and acoustic techniques to record frog species. 

(ii) Pitfall traps associated with drift fence: An X-shaped drift fence with pitfall traps, consisting of 5-m long segments, was used in this study (Malonza et al., 2011). This is a modification of the single array design used by Corn (1994). A drift fence (30 cm high) was stapled vertically onto wooden stakes or pegs. The pitfall traps consisted of 10-L plastic buckets flush to the ground, with every trap array having five buckets in total. The traps were normally opened and checked for five nights and were mostly used for detection of small, primarily nocturnal, crawling herpetofauna not easily detected through other methods. This method was only used in four sites within Mukurumudzi Dam.

(iii) Opportunistic encounters: This qualitative method involved recording all species found or encountered opportunistically for every site outside of the ordinary sampling period of the two standard methods mentioned above. Data from other surveys outside the systematic surveys were also included.

### Shimba Hills National Reserve

The Shimba Hills National Reserve is the main protected area in this ecosystem and is comprised of different habitat types, including forests, grasslands, woodlands, bushlands, and wetlands (rivers and swamps). It is managed by the Kenya Wildlife Service (KWS) in collaboration with the Kenya Forest Service (KFS). The following sites were sampled during this study: Makadara Forest (S04^°^14.253′; E039^°^23.742′; 426 m a.s.l.); Longo-Mwagandi Forest (S04^°^14.264′; E039^°^25.566′; 398 m); Mwele Forest (S04^°^17.198′; E039^°^21.766′; 334 m); Kaya Kwale Forest (S04^°^10.888′, E039^°^26.737′; 417 m); Mwele stream spring (S04^°^16.323′, E039^°^22.214′; 329 m); Shimba Hills Lodge wetlands (S04^°^11.559′; E039^°^25.555′; 290 m); Mwandabara 1 water pump station roadside wetlands (S04^°^10.854′; E039^°^25.158′; 159 m); and Sheldrick Falls (S04^°^17.098′; E039^°^25.849′; 146 m). 

### Forest reserves

The forest reserves are managed by the Kenya Forest Service (KFS), formerly the Forest Department (FD), which allows local communities to harvest sustainably some forest products, such as dead fuelwood. The following sites were sampled during this study: Gongoni Forest (S04^°^24.176′, E039^°^27.604′; 42 m); Gongoni Forest pond (S04^°^25.778′, E039^°^27.755′; 24 m); and Buda Forest (S04^°^ 25.734′, E039^°^ 24.292′; 100 m). 

### Kaya forests

Kayas are sacred sites that owe their continued protection to the long-standing and environmentally friendly Mijikenda community traditions, taboos, beliefs, and cultures. The Mijikenda (nine sub-tribes) includes Giriama, Chonyi, Rabai, Ribe, Kauma, Kambe, and Jibana of Kilifi County along the north coast of Mombasa plus Digo and Duruma of Kwale County along the south coast. These traditions regulate access and conduct within these forests, threatening dire punishment from the spirit world for those who flout the rules (Githitho, 2003). The Kayas occur on hill tops, ridges, valleys, or along coastal beach fronts. Because of their cultural values to the community, most Kayas are protected as monuments under the National Museums and Heritage Act, 2006, and placed under the management of the National Museums of Kenya (NMK). In addition, some are protected as forest reserves and managed by the KFS, but all are managed by the local community Kaya elders in collaboration with either the NMK or KFS. In addition, several Kayas, namely Kaya Giriama (Fungo), Rabai Kayas (Mudzimuvya, Bomu, and Fimboni), Kaya Kambe, Kaya Jibana, Kaya Ribe, Kaya Kauma (all in Kilifi), and Duruma Kayas (Gandini and Mtswakara) in Kwale were listed as World Heritage sites by United Nations Educational Scientific and Cultural Organizatio (UNESCO) in 2008. This was based on their roles as living legacies of the local people’s history, culture, and religion. Currently, however, many Kayas are now highly degraded because the youthful generations disregard these traditions. The following are the Kwale Kaya forests sampled in this study (only during the day): Kaya Kinondo (S04^°^23.588′; E039^°^32.810′; 8 m); Kaya Ukunda (S04^°^18.783′; E039^°^34.047′; 26 m); Kaya Tiwi (S04^°^15.280′; E039^°^35.824′; 20 m); Kaya Muhaka (S04^°^19.941′; E039^°^31.054′; 50 m); Kaya Waa (S04^°^12.131′; E039^°^36.827′; 25 m); Kaya Sega (S04^°^033.254′; E039^°^04.745′; 74 m); Kaya Lunguma (S04^°^07.570′; E039^°^31.198′; 132 m); Kaya Chonyi (S04^°^03.962′; E039^°^ 31.777′; 52 m); and Kaya Gandini (S04^°^ 02.163′; E039^°^ 30.426′; 153 m). 

### Mukurumudzi Dam

The Mukurumudzi Dam area was initially a human settlement along the Mukurumudzi River just below Shimba Hills town. The residents were resettled to pave way for mineral sands mining and subsequent habitat restoration by Base Titanium Limited. Construction of the dam earth embankment started in 2011, with the dam undergoing initial filling in May 2013. Pre-dam herpetological monitoring in four sites started in July 2012 and continued with post-dam monitoring from June 2013 to June 2017. After the dam was filled, the monitoring sites were divided into upstream and downstream sites. The sites selected were those with substantial remnant indigenous forest patches, assumed to act as species refuges for the once expansive coastal forests. In addition, due to their location at the edge of the dam, all sites have wetlands for night frog sampling. The major aim was to determine the impacts of the dam on reptile and amphibian composition over time. The following areas were sampled in this study: Upstream sites: Miembeni (S04^°^22.512′, E039^°^25.529′; 56 m); Maumba (S04^°^23.520′, E039^°^25.774′; 82 m); and Nguluku (S04^°^23.967′, E039^°^25.360′; 87 m); Downstream sites: dam-site (S04^°^24.444′, E039^°^26.021′; 37m); and tailings storage facility-Vumbu (S04^°^23.846′, E039^°^27.057′; 43 m). 

*Community sites sampled:* The Mwabanda wetland, which is a wetland within a rice field stream valley (S04^°^26.499′, E039^°^26.399′; 32 m), and Mtawa stream (S04^°^21.217′, E039^°^28.125; 74 m) were sampled in this study.

### Species data

Data on species presence were acquired from literature and past and recent fieldwork, as well as early specimen collections housed at the Herpetology Section of the NMK since 1960s. 

Collected representative voucher specimens were euthanized in a humane manner according to standard protocols. Amphibian specimens were euthanized with MS222 solution and reptiles with pentobarbital solution and then preserved in 10% formalin and stored in 70% ethanol solution. Selected tissue samples were taken and stored in absolute ethanol for later molecular analysis. Color photographs of selected species and their habitats were taken. A 12 Channel Garmin^®^ receiver was used to collect GPS data. The collected voucher materials were deposited at the NMK in Nairobi. 

## RESULTS

### Species richness

Based on recent field collections, records, and past museum specimens, a total of 89 reptile (47 snakes, 39 lizards, 1 terrapin, 1 tortoise, and 1 crocodile) and 38 amphibian species (36 frogs and 2 caecilians) occur in the Shimba Hills ecosystem. In comparison with other herpetofaunal-rich areas in Kenya, the Arabuko-Sokoke Forest has a total of 80 reptile (46 snakes, 31 lizards, 1 terrapin, and 2 tortoises) and 30 amphibian species (all frogs), the Kakamega Forest has 61 reptile (38 snakes, 21 lizards, 1 terrapin, 1 tortoise) and 24 amphibian species (all frogs), and the Taita Hills have 65 reptile (34 snakes, 28 lizards, 1 terrapin, and 2 tortoises) and 26 amphibian species (24 frogs and 2 caecilians) (Supplementary Table 1, available online).

### Species of conservation concern 

We used the IUCN Red List of threatened species to identify species of conservation concern. In addition, we selected other species with a distribution range restricted to coastal forests and the Eastern Arc Mountains of East Africa that were either rare and/or associated with indigenous forests. The Makadara, Longo-Mwagandi, and Mwele forests in the Shimba Hills National Reserve were the specific forest sites where most species of conservation concern were recorded. The entire species list and distribution in different sites is given in Supplementary Table 1 (available online). Makadara Forest species: *Scolecomorphus vittatus* (Boulenger, 1895), single specimen from the NMK Herpetology Collection; *Callulina krefffti* (Nieden, 1911), first collected in 1961 (Loader et al., 2010). Longo-Mwagandi Forest species: *Prosymna semifasciata* (Broadley, 1995), photographed in September 2014; *Kinyongia tenuis* (Matschie, 1892), IUCN Red List Endangered (EN), collected at the main gate forest and last collected in July 1981. Makadara and Longo-Mwagandi forests: *Elapsoidea nigra* (Günther, 1888), IUCN Red List Endangered (EN), also present in Mukurumudzi Dam forest patch; *Cnemaspis africana* (Werner, 1895), not collected in the past; *Philothamnus macrops* (Boulenger, 1895), also present in Mukurumudzi Dam (dam-site and Maumba) and Gongoni Forest. *Melanoseps pygymaeus* (Broadley, 2006), present in Longo-Mwangadi, Makadara, Buda, Gongoni, and Kaya Kinondo forests. *Holaspis laevis* (Werner, 1895), present in Longo-Mwagandi, Gongoni, and Buda forests, an arboreal and agile species first recorded and collected in Kenya in October 2009 in the Kaya Jibana Forest in Kilifi (Malonza & Bauer, 2014). *Lygodactylus broadleyi* (Pasteur, 1995), present in Longo-Mwagandi, Makadara, Gongoni, Buda, Kaya Muhaka, and Kaya Kinondo forests, as well as the Miembeni Forest patch in Mukurumudzi Dam. *Cordylus tropidosternum* (Cope, 1869), collected in Makadara Forest, Kaya Kwale Forest, and Kaya Gandini Forest. *Boulengerula changamwensis* (Loveridge, 1932), IUCN Red List Endangered (EN), a soil burrowing amphibian species present in Longo-Mwangandi, Makadara, and Buda forests, as well as Mukurumudzi Dam sites (Miembeni and Maumba forest patches). *Gastropholis prasina* (Werner, 1904), present in the Shimba Hills National Reserve in the Sheldrick Falls area, and recorded at the main gate forest and in Buda Forest station. Amphibian species that exhibit water-dependent breeding included: *Leptopelis grandiceps* (Ahl, 1929), IUCN Red List Vulnerable (VU), a forest dependent species only present in Mwele Forest stream spring; and reed frogs *Hyperolius rubrovermiculatus* (Schiøtz, 1975) and *Afrixalus sylvaticus* (Schiøtz, 1974), both categorized as IUCN Red List Endangered (EN) and present in wetlands in the Shimba Hills National Reserve (e.g. Shimba Lodge, Mwadabara, Sheldrick Falls, and Mwele Forest stream), Gongoni Forest, Buda Forest, and Mukurumudzi Dam site wetlands. 

The search continues to verify the status of historical records of *Gastropholis vittata* (Fischer, 1886), *Bitis gabonica* (Duméril & Bibroni, 1845), and *Hemidactylus modestus* (Günther, 1894) in the Shimba Hills National Reserve and surrounding area (Spawls et al., 2002). 

### Shimba Hills biogeographical assignment

The Shimba Hills have been historically categorized as part of the coastal forests of eastern Africa. However, below is a comparison of the species so far recorded in the Shimba Hills ecosystem that are endemic to the two different biogeographical zones. 

Eastern Arc Mountains species: Currently there are seven typical Eastern Arc Mountains herpetofaunal species. However, the recorded Eastern Arc Mountain species has been increasing steadily in the last several decades. Initially, the species known from the Shimba Hills National Reserve only included *Kinyongia tenuis* (last collected in August 1981). Recent collections of this species and records of other Eastern Arc endemics such as the snakes *Elapsoidea nigra* and *Prosymna semifasciata*, caecilian *Scolecomorphus vittatus,* and frogs *Callulina kreffti* and *Leptopelis grandiceps* have been made. In addition, there is also an early collection of a pygmy chameleon assignable to *Rhampholeon temporalis* (Matschie, 1892), but it has yet to be re-collected. 

Coastal forest species: Including the endemic *Hyperolius rubrovermiculatus* and *Afrixalus sylvaticus* and *Rieppeleon brevicaudatus* (Matschie, 1892) species, 13 East African coastal forest endemic species have been recorded in the Shimba Hills ecosystem. These include *Hemidactylus barbouri* (Loveridge, 1942), *Hemidactylus mrimaensis* (Malonza & Bauer, 2014), *Lygodactylus mombasicus* (Loveridge, 1935), *Melanoseps pygmaeus*, *Lygosoma pembanum* (Boettger, 1913), *Lygodactylus broadleyi* (Pasteur, 1995), *Letheobia swahilica* (Broadley & Wallach, 2007), *Letheobia lumbriciformis* (Peters, 1874), *Leptotyphlops macrops* (Broadley & Wallach, 1996), and *Boulengerula changamwensis*. This biogeographical analysis of the Eastern Arc and coastal forests of the Shimba Hills species gives a ratio of 7:13. 

## DISCUSSION

The Shimba Hills stand out as the most reptile and amphibian species rich area in Kenya, particularly due to the endemic and near-endemic species. Undoubtedly, the Makadara, Longo-Mwagandi, and Mwele forests within the Shimba Hills National Reserve are key forest areas because they support all currently known rare and unique herpetofaunal species in this ecosystem. Outside the Shimba Hills National Reserve, Mukurumudzi Dam is the best amphibian habitat. Following the creation of the dam, the ongoing habitat rehabilitation and protection efforts have created new habitats for species, while former areas have improved in quality. This agrees with other studies elsewhere on the effects of large water dams on biodiversity (McAllister et al., 2000). Although the Gongoni, Buda, and Kaya forests are highly disturbed and degraded, they also support some unique forest-associated species. This concurs with past findings in the Kitobo Forest in south-eastern Kenya, which was found to act as a refuge for several threatened forest species despite being surrounded by intensive community settlement and agriculture (Malonza et al., 2011).

Using reptiles and amphibians as zoogeographical indicators, there was no clear support of the hypothesis that the Shimba Hills area is biogeographically a typical coastal forest. Our results showed that the Shimba Hills are at a biogeographical crossroads, supporting a good number of typically Eastern Arc as well as East African coastal forest species (Burgess et al., 2007). This observation was also noted in a brief analysis of the Shimba Hills amphibians by Bwong et al. (2014). Given that most of the Eastern Arc endemics are rare and recent species records, it is likely that their numbers might increase with additional sampling. In addition, further analysis of all forms of biodiversity (flora and fauna), including molecular taxonomy, is needed to clearly understand the biogeographic history of the Shimba Hills biodiversity. 

With their low undulating hills and plateaus, unlike the ordinary coastal lowland forests but like the Eastern Arc Mountains, the Shimba Hills present the first break or barrier to the moist rainfall winds from the Indian Ocean maintaining a stable montane forest environment. The proximity of the Shimba Hills to the Indian Ocean has resulted in a stable climate, leading to its diverse and unique herpetofauna. The hilly topography (up to ~430 m) differentiates the Shimba Hills from typical lowland coastal forests like Arabuko-Sokoke. Furthermore, although the humid montane forests are like islands that promote speciation similar to that observed in the Eastern Arc Mountains, most of the species found in the Shimba Hills occur at much lower elevations than typically recorded elsewhere in their range. For example, the Usambara garter snake (*Elapsiodea nigra*) has been recorded below 100 m in the Mukurumudzi Dam basin, whereas in Tanzania it is known from 300–2 000 m (Menegon et al., 2008; Spawls et al., 2002). 

Biogeographically, the Shimba Hills are an important reptile and amphibian area supporting unique and diverse species of conservation concern. While substantial work has been covered in this study, more extensive and intensive fieldwork using all standard herpetofaunal sampling methods covering different seasons and habitat types is highly recommended. 
